# 2-{[(Dimethyl­amino)­methyl­idene]amino}-5-nitro­benzonitrile

**DOI:** 10.1107/S1600536812048866

**Published:** 2012-12-12

**Authors:** Syed Muhammad Saad, Syed Moazzam Haider, Shahnaz Perveen, Khalid M. Khan, Sammer Yousuf

**Affiliations:** a H.E.J. Research Institute of Chemistry, International Center for Chemical and Biological Sciences, University of Karachi, Karachi 75270, Pakistan; b PCSIR Labortories Complex, Karachi, Shahrah-e-Dr. Salmuzzaman Siddiqui, Karachi 75280, Pakistan

## Abstract

The title mol­ecule, C_10_H_10_N_4_O_2_, is almost planar and adopts an *E* configuration of the azomethine [C=N = 1.298 (2) Å] double bond. The benzene ring is attached to an essentially planar (r.m.s. deviation = 0.0226 Å) amidine moiety (N=CN/Me_2_), the dihedral angle between the two mean planes being 18.42 (11)°. The cyano group lies in the plane of the benzene ring [the C and N atoms deviating by 0.030 (3) and 0.040 (3) Å, respectively], while the nitro group makes a dihedral angle 5.8 (3)° with the benzene ring. There are two distinct inter­molecular hydrogen bonds, C—H⋯O and C—H⋯N, that stabilize the crystal structure; the former inter­actions result in centrosymmetric dimers about inversion centers resulting in ten-membered rings, while the later give rise to chains of mol­ecules running parallel to the *b* axis.

## Related literature

For the biological activity of amidine derivatives, see: Sienkiewich *et al.* (2005 ▶); Sasaki *et al.* (1997 ▶). For a related structure, see: Cizak *et al.* (1989 ▶).
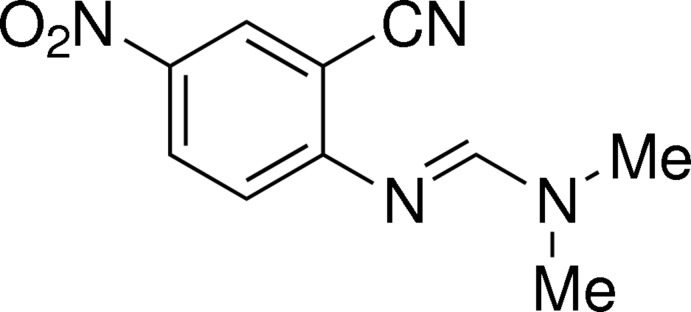


## Experimental

### 

#### Crystal data

C_10_H_10_N_4_O_2_
*M* *_r_* = 218.22
Monoclinic, 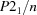

*a* = 7.6496 (11) Å
*b* = 13.0693 (19) Å
*c* = 11.1617 (17) Å
β = 106.475 (3)°
*V* = 1070.1 (3) Å^3^
*Z* = 4
Mo *K*α radiation
μ = 0.10 mm^−1^
*T* = 273 K
0.25 × 0.24 × 0.09 mm

#### Data collection

Bruker SMART APEX CCD area-detector diffractometer
Absorption correction: multi-scan (*SADABS*; Bruker, 2000 ▶) *T*_min_ = 0.976, *T*_max_ = 0.991
6194 measured reflections
1976 independent reflections
1427 reflections with *I* > 2σ(*I*)
*R*_int_ = 0.025

#### Refinement

*R*[*F* ^2^ > 2σ(*F* ^2^)] = 0.046
*wR*(*F* ^2^) = 0.134
*S* = 1.04
1976 reflections
147 parameters
H-atom parameters constrained
Δρ_max_ = 0.18 e Å^−3^
Δρ_min_ = −0.16 e Å^−3^

### 

Data collection: *SMART* (Bruker, 2000 ▶); cell refinement: *SAINT* (Bruker, 2000 ▶); data reduction: *SAINT*; program(s) used to solve structure: *SHELXS97* (Sheldrick, 2008 ▶); program(s) used to refine structure: *SHELXL97* (Sheldrick, 2008 ▶); molecular graphics: *SHELXTL* (Sheldrick, 2008 ▶); software used to prepare material for publication: *SHELXTL*, *PARST* (Nardelli, 1995 ▶) and *PLATON* (Spek, 2009 ▶).

## Supplementary Material

Crystal structure: contains datablock(s) global, I. DOI: 10.1107/S1600536812048866/pv2610sup1.cif

Structure factors: contains datablock(s) I. DOI: 10.1107/S1600536812048866/pv2610Isup2.hkl

Supplementary material file. DOI: 10.1107/S1600536812048866/pv2610Isup3.cml

Additional supplementary materials:  crystallographic information; 3D view; checkCIF report

## Figures and Tables

**Table 1:** Hydrogen-bond geometry (Å, °)

*D*—H⋯*A*	*D*—H	H⋯*A*	*D*⋯*A*	*D*—H⋯*A*
C1—H1*A*⋯O1^i^	0.93	2.48	3.354 (3)	156
C8—H8*A*⋯N1^ii^	0.93	2.61	3.525 (2)	166

Symmetry codes: (i) 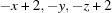; (ii) 
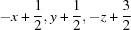
.

**Fig. 1.**
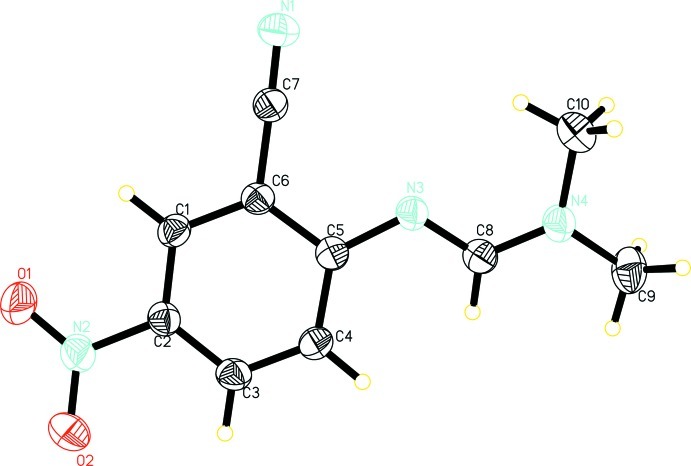
The molecular structure of the title compound with the atom numbering scheme. Displacement ellipsoids are drawn at the 30% probability level. H atoms are presented as small spheres of arbitrary radius.

**Fig. 2.**
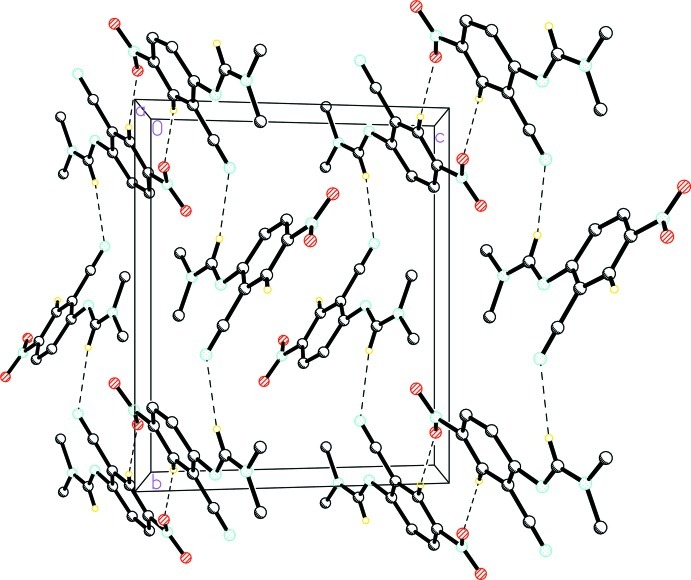
A view of the C—-H···O and C—H···N hydrogen bonds (dotted lines) in the crystal structure of the title compound. H atoms non-participating in hydrogen-bonding were omitted for clarity.

## supplementary crystallographic information

### Comment

The compounds having amidine group (–N═CHNR_2_) in their structures are known to have a wide range of pharmacological properties such as anti-HIV (Sasaki *et al.*, 1997) and anticancer (Sienkiewich *et al.*, 2005). The title compound is also an amidine derivatived we have synthesized in order to evaluate its biological potential and determined its crystal structure that is reported here.

In the title compound (Fig. 1) the benzene ring (C1–C6) is attached with an essentially planar amidine moiety (N3/N4/C8–C10) with r.m.s.d 0.0226 Å; the dihedral angle between the two mean planes being 18.42 (11)°. The atoms C7 and N1 of the cyano group lie in the plane of the benzene ring with deviations 0.030 (3) and 0.040 (3) Å, respectively. The nitro group (N2/O1/O2) makes a dihedral angle 5.8 (3) ° with the benzene ring. The bond distances and angles in the title compound agree very well with the corresponding bond distances and angles reported in a closely related compound (Cizak *et al.*, 1989).

There are two distinct intermolecular hydrogen bonds, C1—H1A···O1 and C8—H8A···N1 that stabilize the crystal structure (Table 2 and Fig. 2). The former interactions result in centrosymmetric dimers about inversion centers resulting in 10-membered rings, while the later give rise to chains of molecules running parallel to the *b*-axis.

### Experimental

5-Nitroanthranilonitrile (45.8 mmol, 7.47 g) was suspended in *N*,*N*-dimethylformamide dimethylacetal (137.4 mmol, 16.5 ml) and the mixture was allow to refluxed for 1.5 h. The progress of the reaction was monitored by thin layer chromatography. After the completion of the reaction, the resulting mixture was cooled to room temperature and refrigerated overnight to obtain yellow crystals. The crystals were filtered, washed with diethyl ether to afford the pure compound (9.4 g, 94% yield). Single-crystal suitable for X-ray diffraction studies were grown from ethanol. All chemicals were purchased by Sigma Aldrich Germany.

### Refinement

The H atoms were positioned geometrically and refined using a riding model, with C—H = 0.93 0.96 Å, for aryl and methyl H-atoms, respectively. The *U*_iso_(H) were allowed at 1.5*U*_eq_(C methyl) or 1.2*U*_eq_(C aryl). A rotating group model was applied to the methyl groups.

### Crystal data

**Table:**

C_10_H_10_N_4_O_2_	*F*(000) = 456
*M**_r_* = 218.22	*D*_x_ = 1.355 Mg m^−^^3^
Monoclinic, *P*2_1_/*n*	Mo *K*α radiation, λ = 0.71073 Å
Hall symbol: -P 2yn	Cell parameters from 1456 reflections
*a* = 7.6496 (11) Å	θ = 2.5–26.3°
*b* = 13.0693 (19) Å	µ = 0.10 mm^−^^1^
*c* = 11.1617 (17) Å	*T* = 273 K
β = 106.475 (3)°	Block, yellow
*V* = 1070.1 (3) Å^3^	0.25 × 0.24 × 0.09 mm
*Z* = 4	

### Data collection

**Table:**

Bruker SMART APEX CCD area-detector diffractometer	1976 independent reflections
Radiation source: fine-focus sealed tube	1427 reflections with *I* > 2σ(*I*)
Graphite monochromator	*R*_int_ = 0.025
ω scan	θ_max_ = 25.5°, θ_min_ = 2.5°
Absorption correction: multi-scan (*SADABS*; Bruker, 2000)	*h* = −9→6
*T*_min_ = 0.976, *T*_max_ = 0.991	*k* = −15→15
6194 measured reflections	*l* = −13→13

### Refinement

**Table:**

Refinement on *F*^2^	Primary atom site location: structure-invariant direct methods
Least-squares matrix: full	Secondary atom site location: difference Fourier map
*R*[*F*^2^ > 2σ(*F*^2^)] = 0.046	Hydrogen site location: inferred from neighbouring sites
*wR*(*F*^2^) = 0.134	H-atom parameters constrained
*S* = 1.04	*w* = 1/[σ^2^(*F*_o_^2^) + (0.0727*P*)^2^ + 0.0566*P*] where *P* = (*F*_o_^2^ + 2*F*_c_^2^)/3
1976 reflections	(Δ/σ)_max_ < 0.001
147 parameters	Δρ_max_ = 0.18 e Å^−^^3^
0 restraints	Δρ_min_ = −0.16 e Å^−^^3^

### Special details

**Table:**

Geometry. All e.s.d.'s (except the e.s.d. in the dihedral angle between two l.s. planes) are estimated using the full covariance matrix. The cell e.s.d.'s are taken into account individually in the estimation of e.s.d.'s in distances, angles and torsion angles; correlations between e.s.d.'s in cell parameters are only used when they are defined by crystal symmetry. An approximate (isotropic) treatment of cell e.s.d.'s is used for estimating e.s.d.'s involving l.s. planes.
Refinement. Refinement of *F*^2^ against ALL reflections. The weighted *R*-factor *wR* and goodness of fit *S* are based on *F*^2^, conventional *R*-factors *R* are based on *F*, with *F* set to zero for negative *F*^2^. The threshold expression of *F*^2^ > σ(*F*^2^) is used only for calculating *R*-factors(gt) *etc*. and is not relevant to the choice of reflections for refinement. *R*-factors based on *F*^2^ are statistically about twice as large as those based on *F*, and *R*- factors based on ALL data will be even larger.

### Fractional atomic coordinates and isotropic or equivalent isotropic displacement parameters (Å^2^)

**Table:**

	*x*	*y*	*z*	*U*_iso_*/*U*_eq_	
O1	1.0056 (2)	0.13577 (12)	1.04462 (17)	0.0870 (6)	
O2	0.8695 (2)	0.25597 (13)	1.11451 (17)	0.0883 (6)	
N1	0.4989 (2)	−0.15217 (12)	0.71893 (17)	0.0660 (5)	
N2	0.8674 (2)	0.18015 (13)	1.04982 (17)	0.0619 (5)	
N3	0.1940 (2)	0.02940 (11)	0.75738 (14)	0.0482 (4)	
N4	−0.1087 (2)	0.05091 (12)	0.65346 (15)	0.0543 (5)	
C1	0.6835 (3)	0.05189 (13)	0.91041 (16)	0.0463 (5)	
H1A	0.7889	0.0151	0.9140	0.056*	
C2	0.6912 (3)	0.14214 (13)	0.97521 (17)	0.0459 (5)	
C3	0.5357 (3)	0.19632 (13)	0.97190 (17)	0.0488 (5)	
H3B	0.5439	0.2565	1.0176	0.059*	
C4	0.3696 (3)	0.16215 (13)	0.90195 (17)	0.0501 (5)	
H4A	0.2657	0.1994	0.9011	0.060*	
C5	0.3520 (2)	0.07166 (12)	0.83108 (16)	0.0426 (4)	
C6	0.5146 (3)	0.01730 (12)	0.83973 (16)	0.0421 (4)	
C7	0.5035 (3)	−0.07743 (14)	0.77211 (17)	0.0490 (5)	
C8	0.0485 (3)	0.08563 (14)	0.72313 (16)	0.0481 (5)	
H8A	0.0558	0.1535	0.7491	0.058*	
C9	−0.2656 (3)	0.11779 (17)	0.6087 (2)	0.0711 (7)	
H9A	−0.2429	0.1815	0.6533	0.107*	
H9B	−0.3713	0.0857	0.6223	0.107*	
H9C	−0.2861	0.1303	0.5210	0.107*	
C10	−0.1270 (3)	−0.05486 (17)	0.6095 (2)	0.0753 (7)	
H10A	−0.0086	−0.0858	0.6275	0.113*	
H10B	−0.1837	−0.0560	0.5209	0.113*	
H10C	−0.2008	−0.0923	0.6510	0.113*	

### Atomic displacement parameters (Å^2^)

**Table:**

	*U*^11^	*U*^22^	*U*^33^	*U*^12^	*U*^13^	*U*^23^
O1	0.0470 (10)	0.0792 (11)	0.1247 (15)	0.0011 (8)	0.0079 (10)	−0.0227 (9)
O2	0.0709 (12)	0.0766 (11)	0.1064 (13)	−0.0137 (8)	0.0075 (10)	−0.0416 (9)
N1	0.0732 (13)	0.0482 (10)	0.0718 (11)	0.0013 (8)	0.0125 (10)	−0.0114 (8)
N2	0.0528 (12)	0.0526 (10)	0.0734 (12)	−0.0048 (8)	0.0068 (9)	−0.0053 (8)
N3	0.0436 (10)	0.0425 (8)	0.0543 (9)	0.0004 (7)	0.0070 (8)	−0.0005 (6)
N4	0.0458 (11)	0.0553 (10)	0.0566 (10)	0.0019 (7)	0.0059 (8)	0.0004 (7)
C1	0.0453 (12)	0.0409 (9)	0.0520 (11)	0.0044 (8)	0.0127 (9)	0.0026 (7)
C2	0.0456 (12)	0.0400 (9)	0.0493 (10)	−0.0033 (8)	0.0087 (9)	0.0021 (7)
C3	0.0559 (13)	0.0358 (9)	0.0518 (11)	0.0005 (8)	0.0103 (9)	−0.0021 (7)
C4	0.0491 (12)	0.0407 (10)	0.0577 (11)	0.0074 (8)	0.0103 (10)	−0.0007 (8)
C5	0.0457 (11)	0.0373 (9)	0.0432 (10)	0.0000 (8)	0.0100 (8)	0.0063 (7)
C6	0.0472 (12)	0.0342 (8)	0.0438 (9)	0.0003 (7)	0.0109 (8)	0.0035 (7)
C7	0.0506 (12)	0.0420 (10)	0.0514 (10)	0.0023 (8)	0.0098 (9)	0.0022 (8)
C8	0.0520 (13)	0.0441 (10)	0.0449 (10)	0.0007 (8)	0.0083 (9)	0.0033 (7)
C9	0.0523 (14)	0.0767 (15)	0.0736 (14)	0.0061 (11)	0.0004 (11)	0.0162 (11)
C10	0.0629 (16)	0.0674 (14)	0.0909 (17)	−0.0132 (11)	0.0141 (13)	−0.0176 (12)

### Geometric parameters (Å, º)

**Table:**

O1—N2	1.222 (2)	C3—C4	1.364 (3)
O2—N2	1.224 (2)	C3—H3B	0.9300
N1—C7	1.138 (2)	C4—C5	1.408 (2)
N2—C2	1.456 (2)	C4—H4A	0.9300
N3—C8	1.298 (2)	C5—C6	1.412 (2)
N3—C5	1.371 (2)	C6—C7	1.440 (2)
N4—C8	1.314 (2)	C8—H8A	0.9300
N4—C9	1.454 (2)	C9—H9A	0.9600
N4—C10	1.460 (2)	C9—H9B	0.9600
C1—C2	1.376 (2)	C9—H9C	0.9600
C1—C6	1.385 (2)	C10—H10A	0.9600
C1—H1A	0.9300	C10—H10B	0.9600
C2—C3	1.375 (3)	C10—H10C	0.9600
			
O1—N2—O2	123.09 (18)	C4—C5—C6	116.26 (16)
O1—N2—C2	118.95 (17)	C1—C6—C5	122.42 (16)
O2—N2—C2	117.96 (17)	C1—C6—C7	119.09 (16)
C8—N3—C5	119.04 (15)	C5—C6—C7	118.50 (16)
C8—N4—C9	121.57 (17)	N1—C7—C6	178.4 (2)
C8—N4—C10	120.69 (17)	N3—C8—N4	122.92 (17)
C9—N4—C10	117.54 (17)	N3—C8—H8A	118.5
C2—C1—C6	118.24 (17)	N4—C8—H8A	118.5
C2—C1—H1A	120.9	N4—C9—H9A	109.5
C6—C1—H1A	120.9	N4—C9—H9B	109.5
C3—C2—C1	121.29 (17)	H9A—C9—H9B	109.5
C3—C2—N2	119.53 (16)	N4—C9—H9C	109.5
C1—C2—N2	119.18 (17)	H9A—C9—H9C	109.5
C4—C3—C2	120.32 (17)	H9B—C9—H9C	109.5
C4—C3—H3B	119.8	N4—C10—H10A	109.5
C2—C3—H3B	119.8	N4—C10—H10B	109.5
C3—C4—C5	121.44 (17)	H10A—C10—H10B	109.5
C3—C4—H4A	119.3	N4—C10—H10C	109.5
C5—C4—H4A	119.3	H10A—C10—H10C	109.5
N3—C5—C4	127.10 (17)	H10B—C10—H10C	109.5
N3—C5—C6	116.62 (15)		
			
C6—C1—C2—C3	0.9 (3)	C3—C4—C5—N3	−179.98 (17)
C6—C1—C2—N2	−179.79 (16)	C3—C4—C5—C6	1.8 (3)
O1—N2—C2—C3	−174.48 (18)	C2—C1—C6—C5	0.7 (3)
O2—N2—C2—C3	5.1 (3)	C2—C1—C6—C7	−179.46 (16)
O1—N2—C2—C1	6.2 (3)	N3—C5—C6—C1	179.62 (15)
O2—N2—C2—C1	−174.19 (18)	C4—C5—C6—C1	−2.0 (3)
C1—C2—C3—C4	−1.1 (3)	N3—C5—C6—C7	−0.2 (2)
N2—C2—C3—C4	179.63 (17)	C4—C5—C6—C7	178.12 (16)
C2—C3—C4—C5	−0.4 (3)	C5—N3—C8—N4	−179.43 (17)
C8—N3—C5—C4	17.3 (3)	C9—N4—C8—N3	−175.20 (18)
C8—N3—C5—C6	−164.50 (16)	C10—N4—C8—N3	−0.5 (3)

### Hydrogen-bond geometry (Å, º)

**Table:**

*D*—H···*A*	*D*—H	H···*A*	*D*···*A*	*D*—H···*A*
C1—H1*A*···O1^i^	0.93	2.48	3.354 (3)	156
C8—H8*A*···N1^ii^	0.93	2.61	3.525 (2)	166

Symmetry codes: (i) −*x*+2, −*y*, −*z*+2; (ii) −*x*+1/2, *y*+1/2, −*z*+3/2.
